# The Power of Single-Cell RNA Sequencing in eQTL Discovery

**DOI:** 10.3390/genes13030502

**Published:** 2022-03-12

**Authors:** Maleeha Maria, Negar Pouyanfar, Tiit Örd, Minna U. Kaikkonen

**Affiliations:** A. I. Virtanen Institute for Molecular Sciences, University of Eastern Finland, 70211 Kuopio, Finland; maleeha.maria@uef.fi (M.M.); negar.pouyanfar@uef.fi (N.P.); tiit.ord@uef.fi (T.Ö.)

**Keywords:** genetics, single cell, eQTL, *cis*-eQTL, transcription

## Abstract

Genome-wide association studies have successfully mapped thousands of loci associated with complex traits. During the last decade, functional genomics approaches combining genotype information with bulk RNA-sequencing data have identified genes regulated by GWAS loci through expression quantitative trait locus (eQTL) analysis. Single-cell RNA-Sequencing (scRNA-Seq) technologies have created new exciting opportunities for spatiotemporal assessment of changes in gene expression at the single-cell level in complex and inherited conditions. A growing number of studies have demonstrated the power of scRNA-Seq in eQTL mapping across different cell types, developmental stages and stimuli that could be obscured when using bulk RNA-Seq methods. In this review, we outline the methodological principles, advantages, limitations and the future experimental and analytical considerations of single-cell eQTL studies. We look forward to the explosion of single-cell eQTL studies applied to large-scale population genetics to take us one step closer to understanding the molecular mechanisms of disease.

## 1. Introduction

The studies of expression quantitative trait loci (eQTLs) offer insight into the molecular mechanisms of genetic variants that are associated with complex diseases. By definition, eQTLs are divided into two types: *cis*- and *trans*-: *cis*-eQTLs are the genomic sequence variants located within a distance cutoff (for example, 1Mb upstream or downstream) of a target gene (the ‘eGene’) (a gene that has an associated eQTL) and correlate with its expression. Any eQTLs lying outside this genomic window are thought to indirectly regulate gene expression and are considered *trans*-eQTLs [1]. For eQTL mapping, high-throughput RNA sequencing (RNA-seq) has largely replaced microarray techniques due to better specificity and sensitivity as well as the capacity to detect novel transcripts, splice junctions and allele-specific gene expression [2].

To date, nearly all eQTL studies have been conducted on bulk RNA samples, where the RNA is collected from millions of lysed cells within a tissue or other biological sample. In a ‘bulk RNA-Seq’ experiment, the gene expression thus represents an average expression across all cells in a sample. As a derivative of RNA sequencing, single-cell RNA (scRNA) sequencing has emerged as technique for gene expression quantification in single cells [3]. The benefits of scRNA-Seq lie in its capacity to profile cellular heterogeneity, cell-type-specific gene expression and identify rare cell types. In this review, we discuss the differences in the bulk and scRNA-Seq methods for eQTL analysis and summarize the current literature in the field, highlighting the benefits and limitations of single-cell-based approaches. We anticipate that single-cell eQTL analysis on a population scale will likely become mainstream in the next few years.

**Figure 1:**
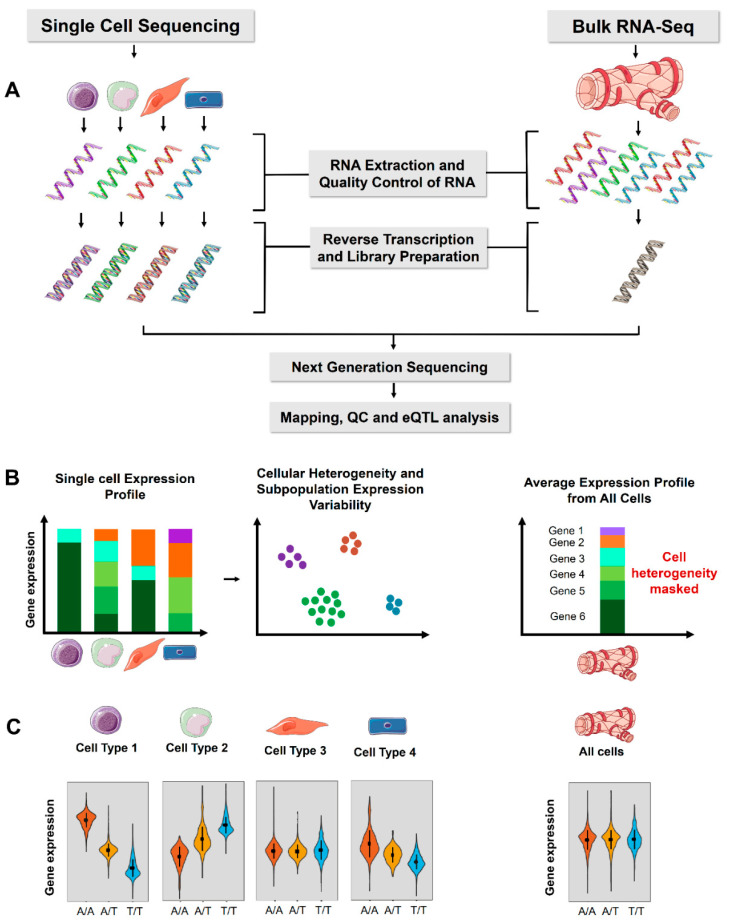
Comparison of single-cell sequencing and bulk RNA-Seq for eQTL analysis. (**A**) The experimental workflow for single-cell and bulk RNA-Seq. (**B**) Single-cell RNA sequencing expression profile includes cellular heterogeneity and expression variability of each cell separately, whereas bulk RNA-Seq represents an average of all the cells in a tissue and cellular heterogeneity cannot be estimated. ScRNA-Seq also allows estimation of variability in gene expression across individual cells. (**C**) Violin plot of an example gene expression for a *cis-*eQTL. The variant is associated with significant allele specific gene expression in individual cell types (left panel) but are masked in bulk tissue analysis. The tissue and cell images were adapted from Servier Medical Art, licensed under a Creative Commons Attribution 3.0 Generic License.

eQTL analysis using scRNA-seq is a relatively new approach and only a dozen studies are available [4,5,6,7,8,9,10,11,12,13,14,15,16]. These studies show diverse applications of scRNA-seq in identification of the quantitative effects of genetic variants or loci using purified cell types [4,5], induced pluripotent stem cells (iPSCs) [6,9,11,13] or whole organisms [10] and to study population ancestry and cell type specific response to an environmental stimulus such as viral infection [12]. In the following sections, we review the published sc-studies for of *cis-*eQTL analysis, and compare the results to bulk RNA-Seq based analysis (Figure 1; Table 1). Altogether, these studies highlight the power of scRNA-Seq in determining cell type specific effects that are not evident in bulk RNA-seq analyses.

## 2. Literature Review

### 2.1. scRNA-Seq in cis-eQTL Analysis of PBMCs

**Figure 2:**
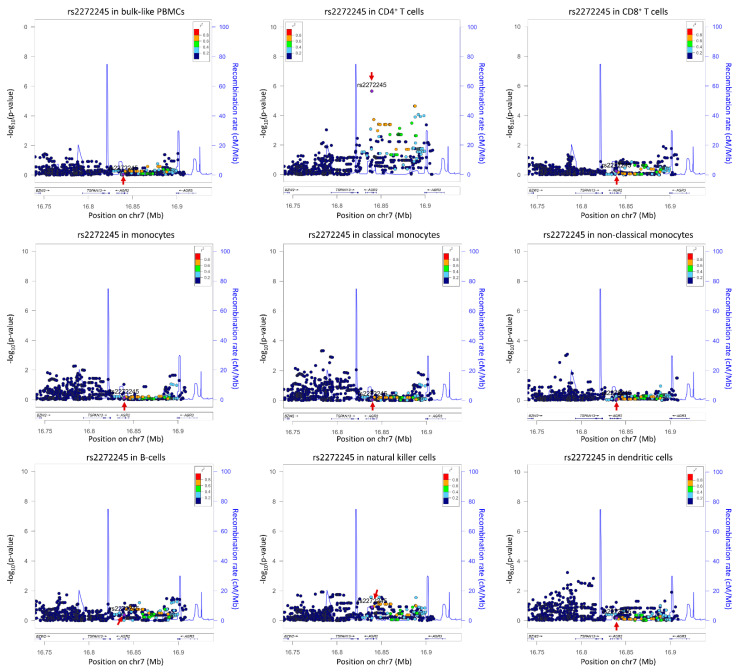
Cell-type-specific locus zoom plot for rs2272245 using summary statistics from [4]. Arrows indicate the SNP rs2272245, a *cis*-eQTL significantly effecting *TSPAN13* expression in CD4^+^ T cells only (*p* = 2.21 × 10^−6^). The number of cells per cell type are as follows: bulk-like PBMCs 25,291; CD4^+^ T cells 13,961; CD8^+^ T cells 4350; monocytes 2630, where classical monocytes 2175 and non-classical monocytes 455; B-cells 835; natural killer cell 2908; dendritic cells 379. Plots were drawn using LocusZoom suite on http://locuszoom.org/ (accessed on 3 February 2022).

The proof of concept for cell-type-specific identification of eQTLs was already shown in 2013, in a study based on 1440 cells from 15 HapMap lymphoblastoid cell lines that measured the expression of 92 genes using highly parallel qPCR [17]. However, the first genome-wide studies using droplet-based scRNA-Seq methods came out in 2018 and made use of the peripheral blood mononuclear cells (PBMCs) that, due to their ease of extraction, have served as a model for the first single-cell sequencing studies. To this end, Kang et al. profiled eQTLs from 78,000 PBMCs from 23 donors that represented eight major immune cell populations [16]. In total, they found 32 *cis-*eQTLs of which 22 were cell-type specific. A comparison with cell-type-specific expression from previous bulk CD14^+^ monocytes, CD4^+^ T cells and lymphoblastoid cell lines revealed a stronger SNP-gene association within similar cell types than across different cell types, e.g., despite ubiquitous expression of CD52 in leukocytes, its associated *cis-*eQTL was identified only in monocytes. The same year, another study by van der Wijst et al. performed cell-type-specific eQTLs identification using scRNA-seq of 25,000 peripheral blood mononuclear cells (PBMCs) from 45 Dutch donors in Lifelines Deep cohort (Table 1) [4,18]. The authors aggregated similar cells from each donor into a broader classification of six categories: CD4^+^ T cells, CD8^+^ T cells, NK cells, monocytes, B cells and DCs [4]. They compared the sc-*cis-*eQTL profiles with two previous bulk studies, namely RNA-Seq (*n* = 2116) [19] and DeepSAGE (*n* = 94) [20], and found 1% to 8% overlap, respectively. The low overlap could reflect the difference in the cellular composition of samples, i.e., PBMCs vs. whole blood and low 3′ sequencing depth achieved in the bulk RNA-Seq studies. They discovered 379 *cis-*eQTLs (287 genes), of which 331 (249 genes) were significant in the bulk-like analysis of PBMC eQTLs (average expression of all cells per gene) and 48 *cis-*eQTLs (38 genes) that were only detected in specific cell types. Interestingly, a larger fraction (78%) of the bulk-like eQTLs were associated with the same SNP in whole blood RNA-seq eQTL dataset, compared to only 60% of the cell-type-dependent eQTLs. This suggested that cell-type-specific signals are diluted in a bulk RNA-seq dataset. For example, due to the low expression of *TSPAN13* in abundant CD4^+^ T cells, the *cis-*eQTL rs2272245 was not identified in the bulk RNA-seq dataset [19] and was only found in scRNA-seq analysis. This shows that bulk RNA-seq based *cis-*eQTL analysis loses power in identification of cell-type-specific loci affecting lowly expressed genes (Figure 2). This study also highlighted another advantage of the scRNA-Seq is to determine co-expression networks on an individual donor basis. They demonstrated that true gene correlations were more evident in their 45 samples than in large-scale bulk RNA-Seq datasets of thousands of individuals. The cell-type-specific scRNA-seq data can thus also be used to identify SNPs altering co-expression relationships, i.e., co-expression QTLs, and to interrogate the directionality and context specificity of networks [4,21].

Recently, scRNA-seq was also used to identify the effects of genetic ancestry on susceptibility to viral infection among individuals of European and African descent [12]. The authors used PBMCs from 90 donors that were either non-infected or infected with influenza A virus (IAV). In total, scRNA-seq data from 255,731 single cells were generated and eight distinct immune cell types were categorized into five clusters CD4^+^ T cells, CD8^+^ T cells, B cells, natural killer (NK) cells, and monocytes (Table 1). *cis-*eQTLs were mapped across all cell types and treatments, and 2234 eGenes had at least one eQTL within 100 kb distance. Importantly, 45% variants were shared across all cell types and conditions, 13–24% of the eGenes were detected in only one condition and only 29 genes were only detectable after IAV infection. Moreover, over >50% of the population differences were explained by differences in the frequency of *cis-*regulatory variants. Together, these single cell results provided the first evidence that most of the ancestry effects on the immune response to environmental stimulus, in this case viral infection, could be cell-type specific.

### 2.2. scRNA-Seq in Identifying cis-eQTL as Spatiotemporal Regulatory Elements in iPSCs

Cellular function is under the control of genetic variants; however, evaluating the effects of these variants in primary cells and in development is challenging. Human iPSC technology provides a useful strategy for estimating these effects during differentiation and to study the molecular mechanisms of diseases in the relevant human cell types. To this end, scRNA-seq technology has been recently explored for the evaluation of cell-type-specific effects of genetic variation during early human development and differentiation (Table 1) [6,9,11,13]. In an elegant study by Cuomo et al., the authors derived 126 iPSC cell lines from 125 donors in HipSci project [22], harvested the cells immediately before differentiation (iPSCs), and at mesendoderm and definitive endoderm stages of differentiation (Table 1) [9]. These cells were sorted and sequenced using Smart-seq2 [23] to generate a final dataset comprising of 36,044 cells with 11,231 genes expressed and 4546 variable genes identified at 10% FDR. *cis-*eQTL analysis of the iPSCs, mesendoderm and definitive endoderm stages identified eQTL for 1833, 1702 and 1342 genes, respectively, among the ~10,000 expressed genes, of which over 30% were specific to a single stage. Moreover, 349 eQTL variants identified during differentiation stages were novel and not previously identified in bulk RNA-Seq from iPSCs or GTEx (Genotype-Tissue Expression) tissues [24]. Furthermore, they demonstrated that expression and eQTL dynamics result in different patterns of allelic expression and illustrated that cis regulatory sequence variation can modulate the timing of expression changes in response to differentiation. For example, VAT1L expression was found repressed during differentiation but repression of the different alleles follows a different timing. This study also took advantage of the resolution provided by single-cell data for co-expression network detection, allowing genotype by environment (GxE) interaction analysis for selected gene-expression modules associated with cell cycle and metabolism. They found that 668 eQTLs were associated with at least one environmental factor, of which 55% had no evidence for a role in differentiation. The authors concluded that although a comparison of eQTL analysis using scRNA-seq with bulk RNA-seq data showed that bulk RNA-seq is more powerful in eQTL identification (*n* = 961, *p* < 5 × 10^−8^), scRNA-Seq proved superior in detailed characterization of eQTLs in a spatiotemporal context, i.e., in recognizing the role of 872 dynamic eQTLs during various stages of differentiation cell cycle or environment-induced cell states.

The use of the same HipSci resource [22] was recently extended to study the differentiation of 215 iPSC lines to midbrain neural fate by mapping eQTLs at three developmental stages: progenitor-like, young neurons, and more mature neurons [6]. scRNA-seq profiles were generated from over 1 million cells generating 26 clusters of 12 distinct cell types. *cis-*eQTLs were mapped for aggregated expression from each donor in main cellular populations identifying a total of 4828 eGenes (Table 1). An overlap of eQTL signals identified in this study with GTEx brain tissue revealed 50% concordance, a brain-specific eQTL replication rate of 10−20%, and demonstrated that, as the tissue matures, the number of shared eQTLs among these datasets increases. Overall, 2366 novel and unique eQTLs were identified in this study. Finally, a colocalization analysis between 25 GWAS traits consisting of neurodegenerative disorders, or conditions related to behavior and intelligence suggested that cell-type-specific colocalization analysis could be more powerful than bulk RNA-seq-based colocalization. Altogether this study demonstrated that the identification of cell type specific eQTLs at distinct time points in development allows the discovery of novel regulatory relationships.

scRNA-Seq has also been used to study the role of variance eQTLs (vQTL) in human development and disease using 7585 iPSCs derived from 54 Yoruba individuals (Table 1) [11]. This generated a dataset with expression profile of 9957 genes from 5597 cells of 53 participants. Altogether, 235 single cells eQTLs were identified of which 79% replicated in bulk data and 80% of bulk eQTLs replicated in single-cell data. Still, down sampling of the bulk RNA-Seq to the same number recovered over one thousand more eQTLs suggesting that increased experimental noise of the scRNA-Seq leads to lower power of discovery. They also identified five vQTLs that could alter the variance of expression independently of the expression mean but explain less phenotypic variance than eQTLs. This again is likely caused by the experimental noise and highlighted the need for larger sample sizes to study variant effects on the dispersion that tend to be smaller than effects on the mean.

A reverse approach to iPSC differentiation recently evaluated the allelic effects of iPSC reprogramming from fibroblasts on single-cell gene expression [13]. Here, human skin fibroblasts were reprogrammed to iPSCs from 79 donors (Table 1). In total 83,985 cells were sequenced using 3′-scRNA-seq including 19,967 iPSCs. Based on the activity of regulating transcription factors, they were able to classify fibroblasts into six types (SIX5^+^, HOXC6^+^, ATF1^+^, TEAD2^+^, KLF10^+^ and RXRB^+^) and iPSCs into four types (HIC2^+^, ATF2^+^, BRF2^+^ and CEBPG^+^). In addition, single-cell *cis-*eQTLs were mapped in six fibroblast cell lines as well as four iPSC lines that were derived from the same participants. Collectively, they identified 46,103 eQTLs in 2985 genes representing 45,503 eQTLs for 2887 genes found in fibroblast cell types and 810 *cis-*eQTLs for 86 genes in iPSC derived cell types. Importantly, the majority of eGenes were predominantly cell type specific and only identified in only one fibroblast type (77.6% of fibroblast eGenes) or one iPSC type (97.2% of iPSC eGenes). Furthermore, the majority of the 283 eGenes that were significant in multiple cell types including the 14 eGenes that overlapped between fibroblasts and reprogrammed iPSCs, were found to be regulated by different genetic loci. A comparison with bulk RNA profile from GTEx cultured fibroblasts [25] showed only 41.1% overlap with the scRNA-seq profile from fibroblasts in this study, but the allelic effects shared the same direction of effect. In line with this, the GTEx cultured fibroblasts exhibited a positive correlation between allelic effect size and the number of cell types for which eGenes were identified. This indicated that cell-type-specific elements are not entirely revealed from bulk RNA-seq data because bulk gene expression profile presents mean expression across all cell types in biological sample under study. Altogether, these data supported a highly cell-type-specific impact of eQTLs in cellular reprogramming and pluripotency.

### 2.3. scRNA-Seq in Determining Whole Organism Genetic Architecture

scRNA-seq has made it possible to sequence many cells simultaneously, analyze cell-type-specific genetic architecture and compare it among different cell types to evaluate how genes regulate the fate of a cell into a particular type. Keeping this as a goal, Ben-David et al. [10], piloted a study that showed scRNA-seq could be successfully applied to whole organism in C. elegans. They cultured C. elegans to second larval stage L2 in F4 generation, dissociated 192,000 F4 worms and isolated the cells and processed using the 10× Genomics scRNA-seq platform. Two parental strains of worms were cultured, processed, and sequenced separately and differentially expressed genes from their eQTL analysis were evaluated in individual cell types and in all cells combined as well as in global manner. A global dataset was used to identify cell types in parental scRNA-seq dataset. In total, they identified 1718 *cis-*eQTLs in 1294 genes across different cell types. A *cis-*eQTL comparison with parental scRNA-seq dataset revealed 870 genes that were differentially expressed; 23% of these had a *cis-*eQTL in the same tissue and 95% of *cis-*eQTLs had same direction of effect as parental. An overlap with a previously published bulk RNA-seq [26] dataset from 200 recombinant and inbred lines from parental strains showed that from 981 identified *cis-*eQTLs, 335 were shared between two studies and had correlated effect sizes (Spearman’s ρ = 0.64, *p* < 2.2 × 10^−16^); 50% of the eGenes detected in multiple cell types were also identified in bulk and 28% of the eQTLs were detected in a single cell type. Finally, a cell type specific analysis of C. elegans nervous system reveled 12,647 neurons in 81 distinct clusters. 163 *cis-*eQTLs and 132 eGenes were identified where 88% were cell-type specific. In a pan-neuronal *cis-*eQTL analysis, 36 of 69 (52%) eQTLs had opposing direction to cell-type-specific eQTLs, confirming a subtype-specific effect. For example, nlp-21 had significantly opposing effects in RIC interneurons and ring interneuron/motor neurons, but no significant effect was found in the pan-neuronal dataset. In conclusion, this study provided direct evidence that the sc-eQTL mapping improves the power to detect cell-type-specific effects but also effects that are specific to subtypes of cells. Although this study was the first one to utilize scRNA-seq in whole-organism eQTL mapping, its applicability in more complex higher organisms could be limited.

### 2.4. Challenges of scRNA-Seq Based eQTL Mapping

**Figure 3:**
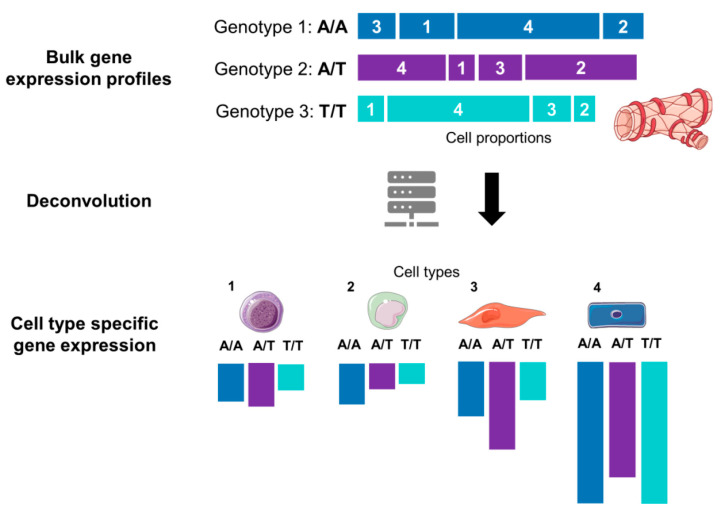
Graphical illustration of the deconvolution of mixed samples. Bulk transcriptomics data for an allele of a given gene are a sum of expression of cell types 1, 2, 3 and 4. After computational deconvolution, cell types are separated, and gene expression of each cell type is estimated considering cell-type proportions from a reference dataset (e.g., scRNA-Seq). The tissue and cell images were adapted from Servier Medical Art, licensed under a Creative Commons Attribution 3.0 Generic License.

The first pioneering studies outlined above have clearly demonstrated the advantage of scRNA-Seq in identifying cell-type- and cell-state-specific eQTLs. Still, many of these studies demonstrated limited power for eQTL mapping due to lower sample numbers that are largely imposed by the high cost of scRNA-Seq experiments (Table 2). This limitation has been addressed in two ways. First, we have witnessed a growing array of methods for statistical deconvolution of the bulk RNA-Seq data (Figure 3) that allows estimation of cell-type proportions based on prior information from purified cell subpopulation (e.g., scRNA-Seq or FACS quantification of the cell proportions). Different deconvolution methods are now available, as exemplified by DeconRNAseq [27], CIBERSORT [28], CIBERSORTx [29], MuSiC [30], DSA [31], and MMAD [32], these with other available methods have been recently compared and discussed [33,34]. These tools are proving highly useful in reanalysis of both existing and new bulk RNA-Seq datasets to identify and interpret the role of cell type specific eQTLs in complex diseases. Secondly, the optimized design of scRNA-seq experiments can also substantially reduce the costs of population scale cell-type-specific eQTL mapping. Using PBMCs from 120 individuals, Mandric et al. modeled the impact of the number of reads, number of individuals, number of cells, level of sample multiplexing, and cell-type classification accuracy on the power of *cis-*eQTL studies. The authors concluded that statistical power of cell-type-specific eQTL mapping can be maximized by increasing the sample size (~100) and the number of cells per sample while performing low-coverage sequencing of 10,000 reads per cell [14]. They further provided a calculator which can guide the selection of sample size and the number of cells per individual for cell-type-specific eQTL detection with the available budget. Still, the caveat of this approach is the risk of losing information from rare cell types due to low and non-uniform coverage. The best way would be to run power calculations using R or R-based packages [14,35,36]. Current single-cell eQTL power calculations assume a standard linear model, and power for eQTL discovery in specific cell types can be improved by incorporating allele-specific mapped reads [37]. Additionally, experimental variation, as witnessed in iPSC research, will introduce noise that would require much larger sample numbers [11]. Nevertheless, highlighting such experimental considerations will help researchers plan their eQTL mapping experiments in a cost- and time-effective manner in the future.

Another limitation of the scRNA-Seq studies comes from the library configuration. Most of the studies thus far have used the 10× Genomics Chromium platform that sequences the 3′- or 5′-end of mRNA and does not allow the identification of splicing QTLs for isoform detection or deep intronic QTLs. These issues can be addressed by the full-length sequencing approaches such as SMART-seq [15] which, however, comes with a higher cost per cells. High technical noise arising from ribosomal or mitochondrial contamination is another challenge in scRNA-seq data. So far, this caveat has been managed by removing sequencing reads that map to >15–25% mitochondrial reads and >50% ribosomal reads [9,13]. However, in situations where nuclear genome expression is under study, nuclei isolated from purified cells can be used. This was shown in a framework for colocalizing human eQTL with 21 complex traits by Eraslan et al. [5]. Similar methods could prove powerful in *cis-*eQTL analysis and understanding patterns of human disease and development.

High technical noise in scRNA-seq data is still a challenge. It arises due to a difference in sequencing platform, sequencing depth, amplification bias, RNA capture efficiency and dropout events. Current noise reduction methods for scRNA-seq data include correcting for batch effect and normalization of the sequencing data. A recent study comprehensively analyzed 28 noise-reducing methods and tools in 55 scenarios comprising of real and simulated datasets and proposed a guideline to select suitable procedures [38]. The study concluded that not a single method can be selected as generalized approach for all scRNA-seq experiments, selection of an appropriate method needs caution and depends on the study design. For example, the default setting of the mostly commonly used data analysis package Seurat [39] uses a canonical correction analysis model that could cause erroneous mixing in a situation of severe cell composition imbalance. In this case, reciprocal PCA model is recommended. Similarly, linear models are also sensitive to cell population imbalances, and their performance is improved by using cell groups as covariate as in scMerge [38,40]. By unmasking the true biological signals of interest, such methods are expected to also improve the detection of significant *cis*-eQTL associations in the future.

Finally, only a limited number of studies have reported scRNA-seq for eQTL analysis in human disease and development [9,11] indicating a gap in cell-type-specific knowledge about genomic architecture in this context. This also shows an urgent need for large-scale sc studies that is another challenge and can be addressed in consortium-based studies [41].

## 3. Conclusions

As discussed above, scRNA sequencing has created immense opportunities for mapping eQTLs across different cell types, developmental stages and stimuli that could be obscured when using bulk RNA-Seq methods. By decreasing the measurement noise that is introduced due to heterogeneity of cells in bulk RNA profiling, identification of eQTLs associated with rare cell types and specific cell states, including developmental stages, stimulus responsive states and cell cycle phases, can be achieved. In addition to the ability to distinguish the cell-type-specific transcriptome features, scRNA-seq studies have shown great potential for identification of gene regulatory networks and gene–gene interactions and co-regulated genetic features [4]. Moreover, with scRNA data, modeling personal or patient-specific gene networks should be feasible [41]. An unexplored area in scRNA-seq application is organelle-specific expression profiling, which is useful in forensics and phylogenetics. With the decrease in the costs of scRNA-seq based analyses, resources with cell-specific expression profile atlases are becoming reality [42]; however, much larger datasets are still needed for such reference panels. Indeed, consortium based sc studies [41] will increase the power of identification of cell-type-specific eQTLs and genetic variants that affect gene regulatory networks in health and disease manifold. In addition to these clinical implications, studies are needed to optimize technical implications such as bringing wet lab and computational costs down and optimizing methods for statistical analyses.

## Figures and Tables

**Table 1:** Summary of studies that utilized both scRNA-seq and bulk RNA-seq datasets for *cis-*eQTL analysis. The replication of the sc-data in bulk setting within same study shows an overlap of identified signal from 41–79%, indicating power of detecting cell type specific signals within scRNA-seq that are missed in a bulk setting.

Study Parameters		Randolph et al., 2021 [12]		Cuomo et al., 2021 [8]		Jerber et al., 2021 [6]		Neavin et al., 2021 [13]		Cuomo et al., 2020 [9]		Sarkar et al., 2019 [11]		van der Wijst et al., 2018 [4]	
		scRNA-Seq	Bulk RNA-Seq	scRNA-Seq	Bulk RNA-Seq	scRNA-Seq	Bulk RNA-Seq	scRNA-Seq		scRNA-Seq	Bulk RNA-Seq	scRNA-Seq	Bulk RNA-Seq	scRNA-Seq	Bulk-like
Methods	Sample type	PBMCs	PBMCs	iPSCs	iPSCs	iPSCs	iPSCs	FB	iPSCs	iPSCs	iPSCs	iPSCs	iPSCs	PBMCs	PBMCs
	# of donors	90	90	174	87	215	48	79	31	125	108	53	53	45	45
	# of cells or CL	255,731	-	174 (CL)	-	1,027,401	182 (CL)	64,018	19,967	36,044	-	5447	-	25,291	25,291
	cell types	8	-	1	-	12	-	6	4	3	-	-	-	6	6
	Association statistics	LM	LM	LMM *	LMM *	LMM	LMM	LM	LM	LMM	LMM	ZINB	ZINB	SC	SC
	MAF	>0.05	>0.05	>0.1	>0.1	>0.05	>0.05	>0.05	>0.05	>0.05	>0.05	>0.05	>0.05	>0.1	>0.1
	FDR eQTLs	<0.1	<0.1	<0.1	<0.1	<0.05	<0.05	<0.1	<0.1	<0.1	<0.1	<0.1	<0.1	<0.05	<0.05
	FDR SNP-eGene pair	<0.1	<0.1	<0.05	<0.1	<0.05	<0.05	<0.1	<0.1	-	-	<0.1 ^‡‡^	<0.1 ^‡‡^	<0.05	<0.05
	*cis-*distance (kb)	100	100	100	100	250	250	100	100	250	250	100	100	100	100
Results	*cis-*eQTLs	-	-	-	-	15,493	-	45,503	810	4442	-	240	1136	379	331
	eGenes	2234	-	1835	2590	4828	-	2887	86	4470	2908	-	-	287	249
	Replication ^‡‡‡^	-		48% ^‡^ (a)		10–20% (b)		41% (a)		70% (a)		79% (a)		60% (a)	

Abbreviations: CL—cell line; FB—fibroblasts; kb—kilobases; LM—linear model; LMM—linear mixed model; PBMCs—peripheral blood mononuclear cells; SC—Spearman correlation; ZINB—zero-inflated negative binomial distribution; #—number. * Results in mean aggregation setting and LMM outperformed other settings and are therefore used here for comparison. ^‡^ The replication rate raised to 74% in larger all bulk cohort (*n* = 526), number of eGene identified in this dataset was 2448. ^‡‡^ Benjamini–Hochberg procedure. ^‡‡‡^ Percentage of scRNA-seq eQTLs or eGenes identified in a bulk RNA-seq data (a) or bulk eQTLs or eGenes in sc-data (b).

**Table 2:** Example costs of RNA-Seq library prep and sequencing per sample, based on published service price estimates at a university core facility *.

Method	Library Prep Cost/Sample	Sequencing Cost/Sample	No. of Reads (Millions)
Bulk RNA-seq (poly-A)	$260	$125	40
scRNA-Seq (10× Genomics Chromium)	$1610	$1750	450

* https://functionalgenomicscore.ucsf.edu/getting-started (accessed on 16 December 2021).

## Data Availability

Not applicable.
